# THE EFFECT OF DIGITAL INTERACTION WITH FRIENDS AND FAMILY ON OLDER ADULTS’ WELL-BEING: A SYSTEMATIC REVIEW

**DOI:** 10.1093/geroni/igae098.2434

**Published:** 2024-12-31

**Authors:** Ranran He, Destiny Ogle, Jinnan Ren

**Affiliations:** Purdue University, West Lafayette, Indiana, United States; Purdue University, West Lafayette, Indiana, United States; Purdue University - West Lafayette, West Lafayette, Indiana, United States

## Abstract

Contacting friends and family using digital technologies has grown in popularity among older adults. Given the importance of social interaction across the life course, this novel form of interaction may substantially shape their well-being. Existing studies reviewing the effects of technology use on older adults’ well-being were mostly broad in scope, without specifying the age group or the relationships between interactants. This study aims to fill a gap in the literature by systematically reviewing how digital interaction with friends or family shapes older adults’ well-being. We sourced articles from six databases, and the last search was performed in February 2024. Studies were synthesized and analyzed using the narrative approach. All 21 qualifying studies were quantitative in their design. Most studies (10 articles) found positive impacts of digital interaction with family members on well-being, whereas some found negative (4), mixed (3), or null effects (2). Even more conflicting results were found in studies examining the effects of digital interaction with friends; four found positive benefits, five found adverse impacts, and two found no effects. Communication with family members was typically more predictive of well-being than communication with friends. The aggregated measurement of digital contact, the types of digital technology examined, the age range of survey respondents, the sociocultural context, and whether the studies were undertaken during the COVID-19 pandemic may explain these inconsistent findings. Therefore, we suggest that future research incorporate more precise measures of digital interaction with friends and family, more diverse study designs, and explore socio-structural and relational moderators.

